# Towards improving the prognosis of stroke through targeting the circadian clock system

**DOI:** 10.7150/ijbs.88370

**Published:** 2024-01-01

**Authors:** Suliman Khan, Rabeea Siddique, Yang Liu, V. Wee Yong, Mengzhou Xue

**Affiliations:** 1 Department of Cerebrovascular Diseases, The Second Affiliated Hospital of Zhengzhou University, Zhengzhou, China.; 2 Hotchkiss Brain Institute and Department of Clinical Neurosciences, University of Calgary, Calgary, Alberta, Canada.

**Keywords:** Ischemic Stroke (ICH), Circadian clock, Immune system, Treatment and recovery

## Abstract

Rhythmicity of the circadian system is a 24-hour period, driven by transcription-translation feedback loops of circadian clock genes. The central circadian pacemaker in mammals is located in the hypothalamic suprachiasmatic nucleus (SCN), which controls peripheral circadian clocks. In general, most physiological processes are regulated by the circadian system, which is modulated by environmental cues such as exposure to light and/or dark, temperature, and the timing of sleep/wake and food intake. The chronic circadian disruption caused by shift work, jetlag, and/or irregular sleep-wake cycles has long-term health consequences. Its dysregulation contributes to the risk of psychiatric disorders, sleep abnormalities, hypothyroidism and hyperthyroidism, cancer, and obesity. A number of neurological conditions may be worsened by changes in the circadian clock via the SCN pacemaker. For stroke, different physiological activities such as sleep/wake cycles are disrupted due to alterations in circadian rhythms. Moreover, the immunological processes that affect the evolution and recovery processes of stroke are regulated by the circadian clock or core-clock genes. Thus, disrupted circadian rhythms may increase the severity and consequences of stroke, while readjustment of circadian clock machinery may accelerate recovery from stroke. In this manuscript, we discuss the relationship between stroke and circadian rhythms, particularly on stroke development and its recovery process. We focus on immunological and/or molecular processes linking stroke and the circadian system and suggest the circadian rhythm as a target for designing effective therapeutic strategies in stroke.

## Introduction

The exposure of organisms to disrupted events, such as altered day-night cycles and seasonal cycles, can cause changes in behavior and physiology. Adaptation to these environmental cues requires intrinsic regulators, which have long been identified as the circadian system and modulated by the circadian clock 1. This circadian clock contains components including Period circadian homologues (*PER1, PER2, PER3*), Cryptochrome (CRY1, CRY2), circadian locomotor output cycle kaput (*CLOCK*), *NPAS2*, and brain and muscle ARNT-like protein 1(*BMAL1*), which play a critical role in rhythm generation and determine the circadian rhythm across the 24 hour light/dark cycles 2. Circadian disruption can be caused by environmental influences such as shift work or travel across time zones. Chronically disrupted circadian rhythm is known to affect CNS outcomes such as depression and anxiety 3, and it is increasingly implicated in neurological conditions. Moreover, conditions such as stroke have profound immune changes 4, and immune responses and inflammatory processes are influenced by seasonal cycles, and light and dark conditions around the 24-hour clock 5. The severity and outcomes of stroke have also been found to be associated with 24-hour rhythms. For instance, patients with known onset morning stroke have higher in-hospital mortality 6. These observations suggest a link between circadian clock/rhythms and with onset, development, and recovery processes of stroke.

Stroke is a leading cause of death and disability, affecting 13.7 million people and resulting in 5.5 million deaths per year worldwide. It is broadly classified into hemorrhagic stroke (intracerebral hemorrhage and subarachnoid hemorrhage) and ischaemic stroke 7. Hemorrhagic stroke is further classified into intracerebral hemorrhage and subarachnoid hemorrhage. intracerebral hemorrhage constitutes 15-20% of all strokes 7,8 and is caused by bleeding within the brain 7,8. It is particularly catastrophic with a high mortality rate of up to 50% due to prognosis 7,9. Although stroke affects a large number of people across the globe, treatment strategies still need to be discovered or developed. Therefore, it is necessary to understand the molecular mechanisms that could help in developing advanced options for the treatment and prevention of stroke. Deficient blood flow and limited oxygen supply to the brain cause ischemic stroke, while leaky blood vessels or bleeding induces hemorrhagic stroke. Ischemic occlusion creates thrombosis, where the narrowing of blood vessels affects the blood flow and leads to constriction of the vascular chamber and clot formation, thereby causing thrombotic stroke. Decreased blood flow to the brain region also causes embolic stroke, resulting in severe stress and necrosis, followed by plasma membrane disruption, swelling of organelle, and cellular contents leaking into the extracellular environment. In the case of hemorrhagic stroke, internal brain injury causes the rupture of blood vessels or infarction. Intracerebral hemorrhage is associated with the rupture of blood vessels, and abnormal blood accumulation within the brain caused by hypertension and disrupted vasculature 10,11.

Circadian biology is thought to affect the mechanisms associated with susceptibility, progression, recovery, and therapeutic responses in stroke 12. In this article, we review the interconnection between the circadian clock and stroke. We discuss the molecular mechanisms of circadian rhythm and describe that immune cells that affect stroke outcomes are under circadian control. In turn, we discuss that clock genes modulate stroke pathophysiology through immunological responses. Finally, we consider strategies that may improve the prognosis of stroke by affecting the circadian clock. We hope that this review will inspire researchers to uncover the intricate links between the circadian clock and stroke.

## Molecular players of the circadian rhythm in mammals

**Figure 1:**
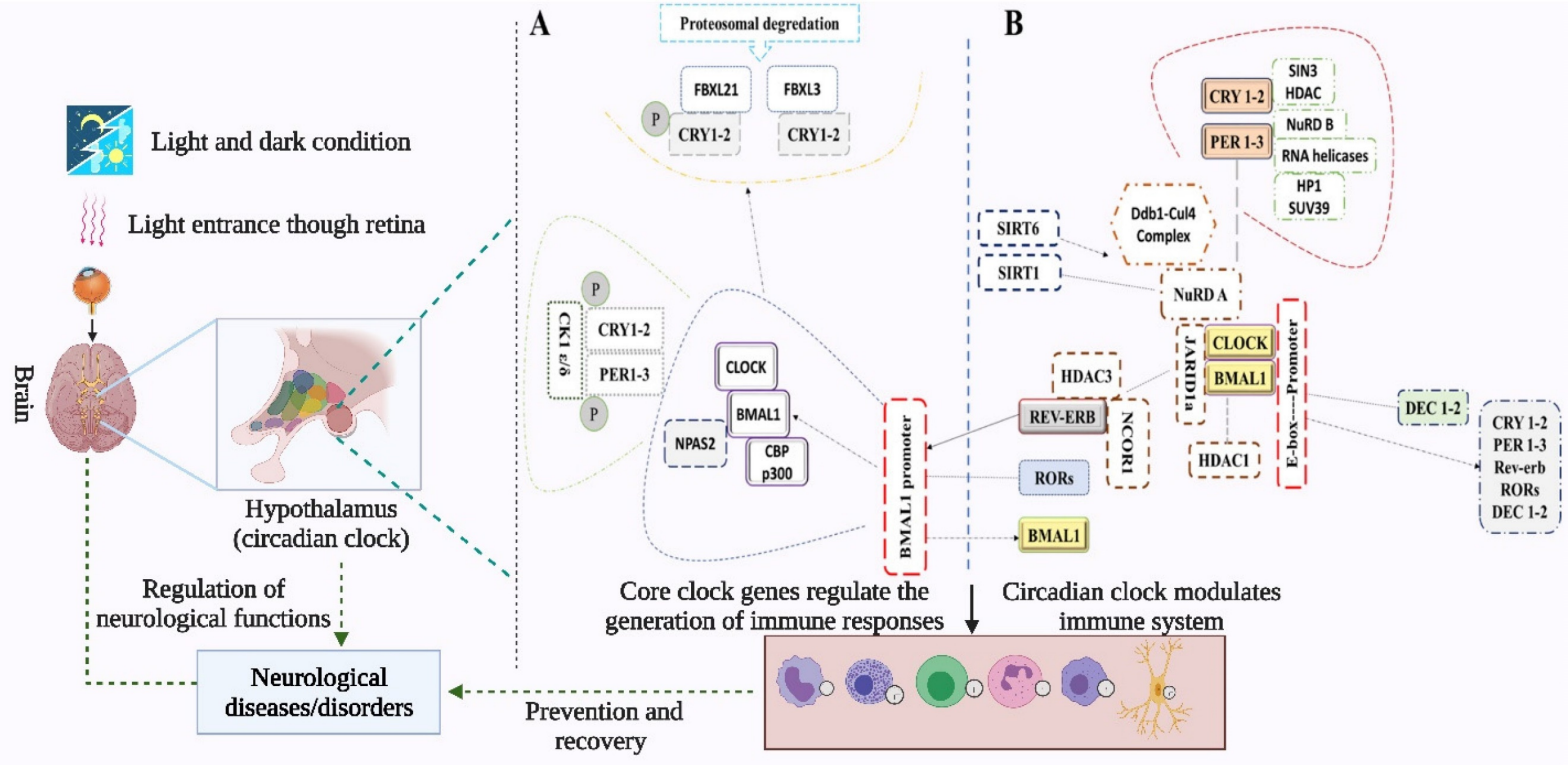
Mechanism of the circadian clock and its regulation of immune cells. Light from environmental sources enters through the retina and interacts with circadian clock genes in the SCN of the hypothalamus 57, to generate rhythms and regulate physiological and immunological functions. The transcription-translation feedback loops in the mammalian molecular clock center around BMAL1 and CLOCK, which direct transcription of other core-clock genes (*PER1*, *PER2,* and *PER3* and *CRY1* and *CRY2*). The complexes generated from PER and CRY proteins translocate into the nucleus to inhibit *CLOCK/BMAL1* transcription, which then represses *PER/CRY* and gets reactivated. The transcription of *RORα* and *REV-ERBα* is activated by CLOCK/BMAL1 complex in a second loop, where the transcription of *BMAL1* is repressed. Next, BMAL1: CLOCK complex is displaced from E-boxes by DEC1 and DEC2 thereby regulating the expression of *PER1*. The immune responses help in the recovery process and prevent the development of severe symptoms of stroke. Immune cells including monocytes, macrophages, mast cells, neutrophils, eosinophils, and natural killer (NK) cells contain intrinsic circadian clocks. Immune functions including phagocytotic and cytotoxic activities, and the release of immune molecules are regulated and modulated by both the central circadian clock of SCN and the intrinsic circadian clocks of the immune cells.

The mammalian molecular clock consists of transcription-translation feedback loops centering around two transcription factors, BMAL1 and circadian locomotor output cycle kaput (CLOCK) 13. BMAL1 and CLOCK heterodimerize and bind to E-box sites to direct transcription of *PER1*, *PER2,* and *PER3* and *CRY1* and *CRY2*. PER and CRY protein complexes subsequently translocate into the nucleus where they inhibit the transcriptional activity of *CLOCK/BMAL1*. *CLOCK*/BMAL1 is reactivated after repressing the transcription of *PER/CRY* through the degradation of PER and CRY proteins, and hence a new cycle begins. CRY1 and CRY2 repress transcription in a vastly different manner at different phases. For instance, CRY2, in association with PER, represses E-box-dependent transcription in the subjective night by displacing CLOCK and BMAL1 from gene promoters, unlike CRY1 which extends this repression into the early morning by interacting with CLOCK and BMAL1 in a PER-independent fashion 14 (Figure 1).

In a second feedback loop, the CLOCK/BMAL1 complex activates the transcription of *RORα* and *REV-ERBα*, which in turn activates and represses *BMAL1* transcription, respectively 15. *RORs* and *rEV-ERBα/β* are themselves clock-controlled through E-box elements within their promoters 16,17. In a third feedback loop, DEC1 and DEC2 proteins displace BMAL1:CLOCK from E-boxes 16,17 and regulate the transactivation of *PER1* 15. Kinases, phosphatases, and ubiquitin ligases modulate the dimerization, subcellular localization, and degradation of PERs and CRYs via post-translational modifications. BMAL1/CLOCK transcriptional activity is modulated by acetylation, phosphorylation, and sumoylation 14. The anatomical location of the brain's circadian clock is the SCN in the hypothalamus, situated directly above the optic chiasm and thus proximal to light input from the eye (Figure 1). SCN is both necessary and sufficient for the generation of circadian rhythms, where the core clock transcription factors *CLOCK* and *BMAL1* are required for the circadian expression of the core clock repressors *PER1* and *PER2* 18. SCN receives direct photic input from a type of photoreceptor cells in the retina termed the intrinsically photoreceptive retinal ganglion cells (ipRGCs). These ipRGCs express a photopigment, melanopsin, that renders them intrinsically photosensitive to short-wavelength irradiation. An emerging theme is that melanopsin-positive ipRGCs are involved in a broad array of nonvisual photic responses in mammals 19,20.

SCN controls behavioral and physiological rhythms through diffusible molecules that are produced by SCN neurons, as well as by targeting other regions of the brain directly. SCN controls peripheral oscillators, and its loss results in the desynchronization of peripheral clocks 2. However, tissue-specific gene expression patterns are likely to be regulated by both local and central mechanisms. The rhythmic gene expression can be driven by both local intracellular clocks and extracellular systemic cues. In addition, complex feedback loops link the circadian clock with rhythmic metabolic networks, integrating these systems in a light-independent manner. Overall, the circadian system's proper functioning requires a combination of local signals, endocrine signaling, temperature, and autonomic innervation of peripheral tissues 19, amongst others.

## A brief introduction to stroke

Ischemic and hemorrhagic strokes are the leading causes of disability worldwide. Ischemic stroke is caused by arterial occlusion and is defined as an infarction of the brain, spinal cord, or retina. It represents ~71% of all strokes. Intracerebral hemorrhage involves spontaneous extravasation of blood into the brain parenchyma and has an incidence of 24.6 per 100,000 persons each year 10,21. It has the greatest morbidity and mortality among all stroke types, with mortality of 31% at 7 days and 59% at 1 year, compared to ischemic stroke of 6.9% at 7 days and 23.6% at 1 year 21. ICH-related brain damage via mechanical destruction of brain tissue is contributed by toxic hemoglobin-released iron and enlarging hematoma that can affect the local cerebral blood flow. Cerebral edema raises intracranial pressure and reduces cerebral perfusion pressure, followed by an inflammatory response, enzyme activation, release of injury mediators, and tissue breakdown and repair. Stroke-mediated brain injury triggers the inflammatory response, enzyme activation, and immunological processes. It is further associated with Matrix Metalloproteinases (MMPs), thrombin, plasmin, and complement proteins that leak from plasma into the brain parenchyma and/or are produced by brain cells. The factors that exacerbate secondary injury have been well-reviewed 8, and they include matrix metalloproteinases (MMPs), iron from hemoglobin, reactive oxygen species, thrombin, plasmin, and complement proteins that leak from plasma into the brain parenchyma and/or produced by brain cells. MMPs and ROS species are critical in stroke development and recovery processes. For instance, MMP-2 (gelatinase A) and MMP-9 (gelatinase B) have the ability to degrade basement membranes, thus gaining major attention from researchers. Earlier studies have indicated that increased levels of MMP-9 correlate with poor neurological outcomes following stroke therapy 22,23. Interestingly, MMP levels are associated with biphasic opening of the blood-brain barrier, therefore, targeting MMPs can be a suitable therapeutic strategy for the treatment of stroke. The association of MMP-2 and MMP-9 with early and late opening of the blood-brain barrier is critical in drug delivery to the brain tissues. On the other hand, reactive oxygen species (ROS) and other oxidants cause oxidative stress, which contributes to the pathogenesis of stroke. Therefore, ROS regulation can help in stroke recovery or treatment. Being a major source of ROS, NADPH oxidases are the main contributors to post-stroke oxidative stress 24. Besides activation of CNS-intrinsic microglia, infiltrated leukocytes in ischemic and hemorrhagic include neutrophils, monocyte-derived macrophages, T lymphocytes, and ILCs. Following brain injury or stroke, activated microglia migrate to the damaged area and release proinflammatory cytokines. Activated microglia induce the production of ROS and thus can contribute to the blood-brain barrier's damage. It is known that the activation of microglia induces the severity of stroke, while its inhibition can protect the brain against stroke 24,25. Concluding, MMP-3 and MMP-9 are produced by microglia, infiltrating inflammatory cells, and astroglia 10,26. Meanwhile, reactive oxygen species (ROS) come from activated microglia and contribute to stroke 27-29.

## The importance of associating Stroke with Circadian system

Circadian rhythm regulates physiological functions and is associated with health hazards; hence normal rhythms are important for health 30. The impact of certain treatment strategies is linked with circadian rhythms and clock responses. For instance, the pharmacokinetics of drugs depend on 24-hour rhythms for effects and safety. Recently, chronotherapy with medication has received much attention. It involves tailored dose timing in accordance with the natural rhythms and/or behavioral patterns of the body, thereby minimizing adverse effects and/or increasing the beneficial effects of medications 31. This strategy can be helpful for diseases with symptoms varying over time such as arthritis, asthma, sleep disorders, depressive disorders, hypertension, myocardial infarction, and congestive heart failure. Therefore, it can also be considered for stroke and related cerebrovascular conditions 32.

**Figure 2:**
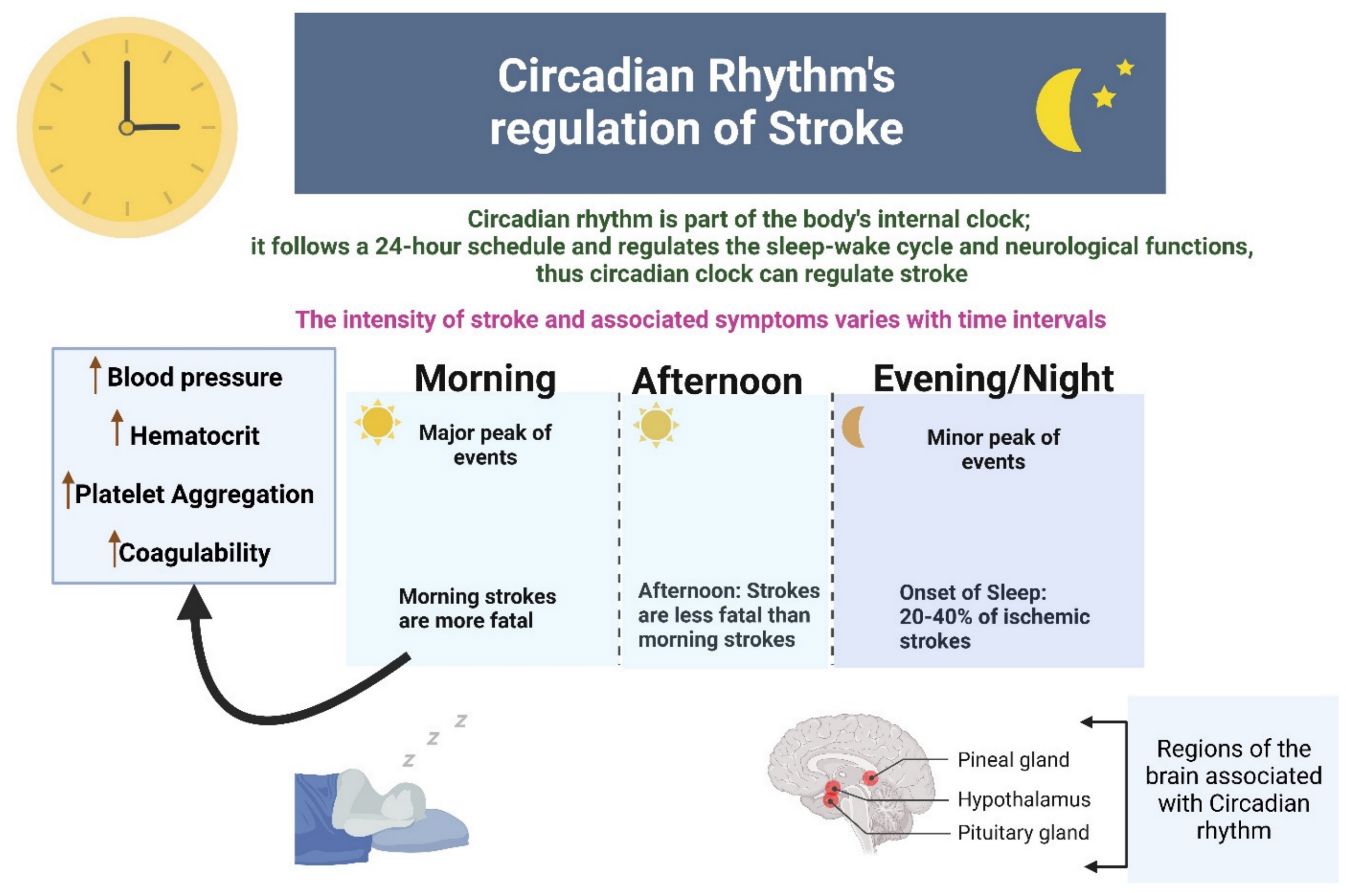
This figure shows that circadian rhythm regulates stroke in the aspects of intensity, fatality and progression. Temporal pattern of stroke events occurs in humans where both ischemic and hemorrhagic strokes have a bimodal pattern with the major and minor peak of events in the morning and evening respectively.

Studies have found that a temporal pattern of stroke events occurs in humans where both ischemic and hemorrhagic strokes have a bimodal pattern with the major and minor peak of events in the morning and evening respectively. 20-40% of ischemic strokes occur at the onset of sleep at night. Morning strokes are more likely to be fatal as compared to afternoon strokes, and this high fatality is related to circadian clock-mediated morning rise in blood pressure, increased hematocrit and platelet aggregation, and hypercoagulability 9 (Figure 2).

Meng et al. investigated the link between stroke and circadian rhythms in rats and observed perturbations in the timing of melatonin secretion in subsequent days after stroke 33. Thus, linking stroke to circadian rhythm can provide new clues to the molecular mechanisms of stroke, and how best to treat stroke for optimal recovery. It is important to investigate the link between stroke and circadian rhythm both at clinical and laboratory levels. Different indicators may interrogate the interdependence of stroke with circadian rhythm. One such biomarker is ARRB1, which was identified as one of the clock-controlled genes expressed in salivary glands. Its circadian profile in the saliva of jetlagged individuals reflects the time lag more than the profile of melatonin 34, suggesting its potential utility as a biomarker in patients with stroke.

Common symptoms of stroke include depression, aggression, and anxiety; these and the risk factors for stroke such as hypertension and cardiac problems 7 can be regulated by circadian rhythm. Moreover, stroke and circadian rhythms both are directly associated with sleep behaviors, sleep and wake onsets, and different neurological activities 3. These details suggest an impact of circadian rhythm on stroke specifically in terms of stroke recovery, but much work is required to establish a causal link between clock-regulated mechanisms and the pathogenesis of, and recovery from, stroke. Moreover, the role of circadian rhythms in the process of recovery should be investigated. Finally, the relationship between the circadian clock and the regulation of immune-responsive cells and genes associated with stroke should be studied. Such studies will be helpful in developing therapeutic strategies for the treatment of stroke that could further enhance the recovery process. For example, neuroprotection is given primary importance in the treatment and recovery of stroke. A number of neuroprotectant strategies have been found effective in rodent models of stroke but failed in clinical trials.

Previous research has indicated that circadian rhythms can contribute to the effects of neuroprotectants in neuroprotection. Esposito et al 35 reported that diurnal rhythms may contribute to the failure of neuroprotectants in humans. The authors tested three different neuroprotective approaches independently, which reduced infarction in inactive phase (daytime) rodent models of stroke but not in active phase (nighttime) rodent models of stroke. Moreover, laser-speckle imaging showed a narrower penumbra of cerebral ischemia in the active-phase mouse model as compared to that of the inactive-phase model. These findings indicate that the influence of circadian rhythm must be considered in neuroprotection for translational studies in stroke.

## Immune cells influencing stroke outcomes are under circadian control

**Figure 3:**
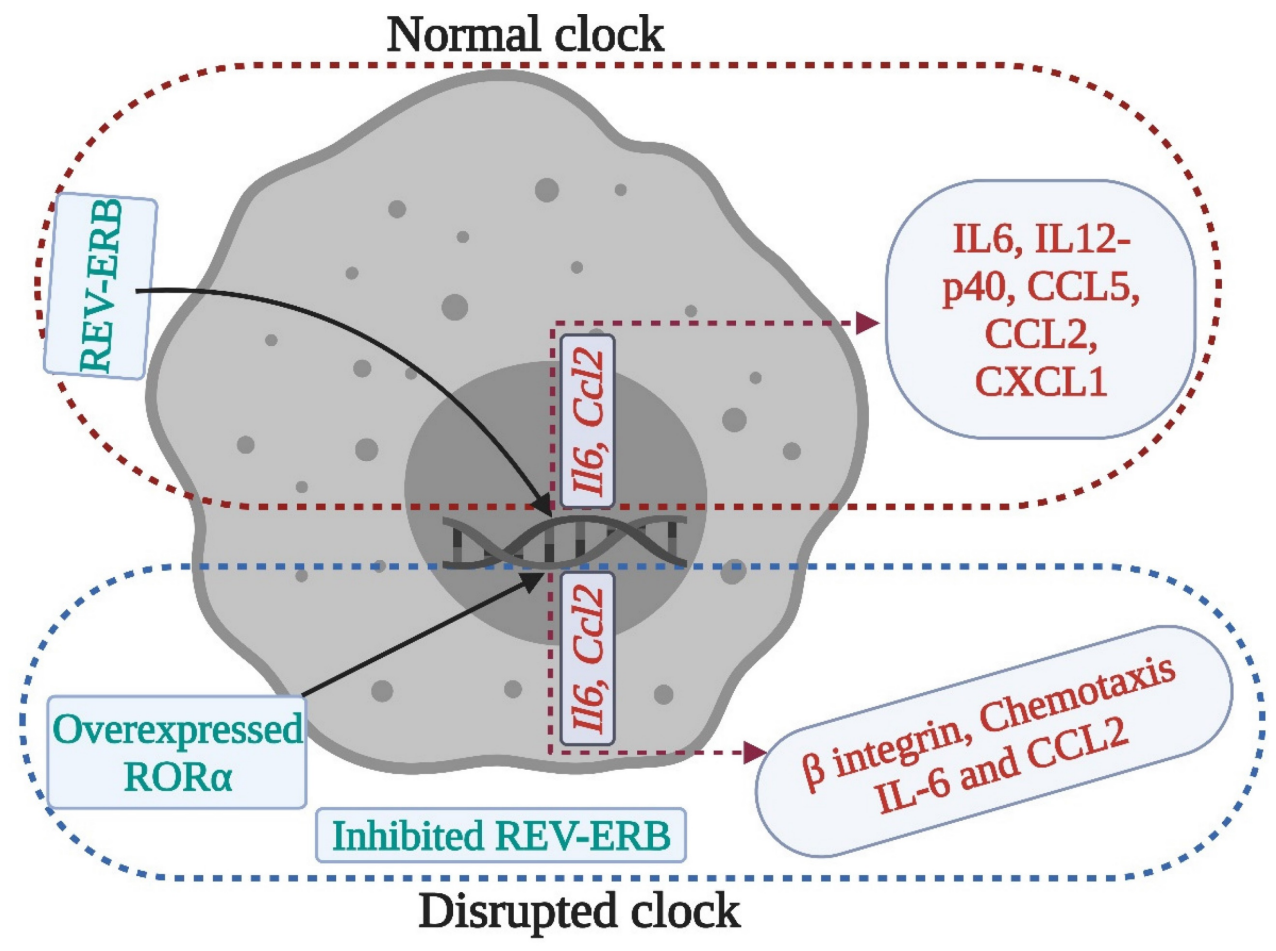
Clock regulation of macrophage immune responses. Clock disruption by REV- ERB deficiency or RORα overexpression in macrophages produces a pro-inflammatory phenotype, where cytokines such as IL-6 and chemokines such as Chemokine ligand (CCL) 2 lose their rhythmic expression. These disruptions may affect the stroke recovery processes.

The involvement of the immune system in secondary injury in stroke, as well as the role of the immune system in recovery, has been well-reviewed 8. The functional and physiological regulations of stroke-associated immune cells are under the control of the circadian system, thus indicating a significant link between stroke and the circadian clock (Figure 2 and Figure 3). Following the stroke, microglia produce reactive oxygen species, cytokines (e.g. IL-1β), MMP-3, and MMP-9, and they contribute to post-ischemic injury but also in its recovery process. Microglia exhibit increased branching of their processes during the dark phase and vice versa in the light phase 36, indicating their association with circadian rhythms. These diurnal morphological variations of microglia are abolished with the disruption of the circadian clock. Moreover, the generation of a specific lysosomal cysteine protease, cathepsin S, from microglia is also under circadian control; this protease affects dendritic spines and synaptic strength 37.

The occurrence and progression of stroke are largely impacted by microglial inflammatory responses, which are tightly controlled by the circadian clock. Thus altered circadian rhythms might predispose individuals to stroke 38. Similarly, macrophages contribute to brain injury following stroke, as they produce pro- and anti-inflammatory cytokines (Figure 3), chemokines, and reactive oxygen species. Macrophages express canonical clock genes including *BMAL1*, *CRY1-2*, *PER1-3*, and *REV-ERBα*, which modulate the production of tumor necrosis factor -α and IL-6. Moreover, the activity of phagocytosis and production of pro-inflammatory cytokines and chemokines are under circadian control, regulated in part by *BMAL1* and *REV-ERBαs* 36.

**Figure 4:**
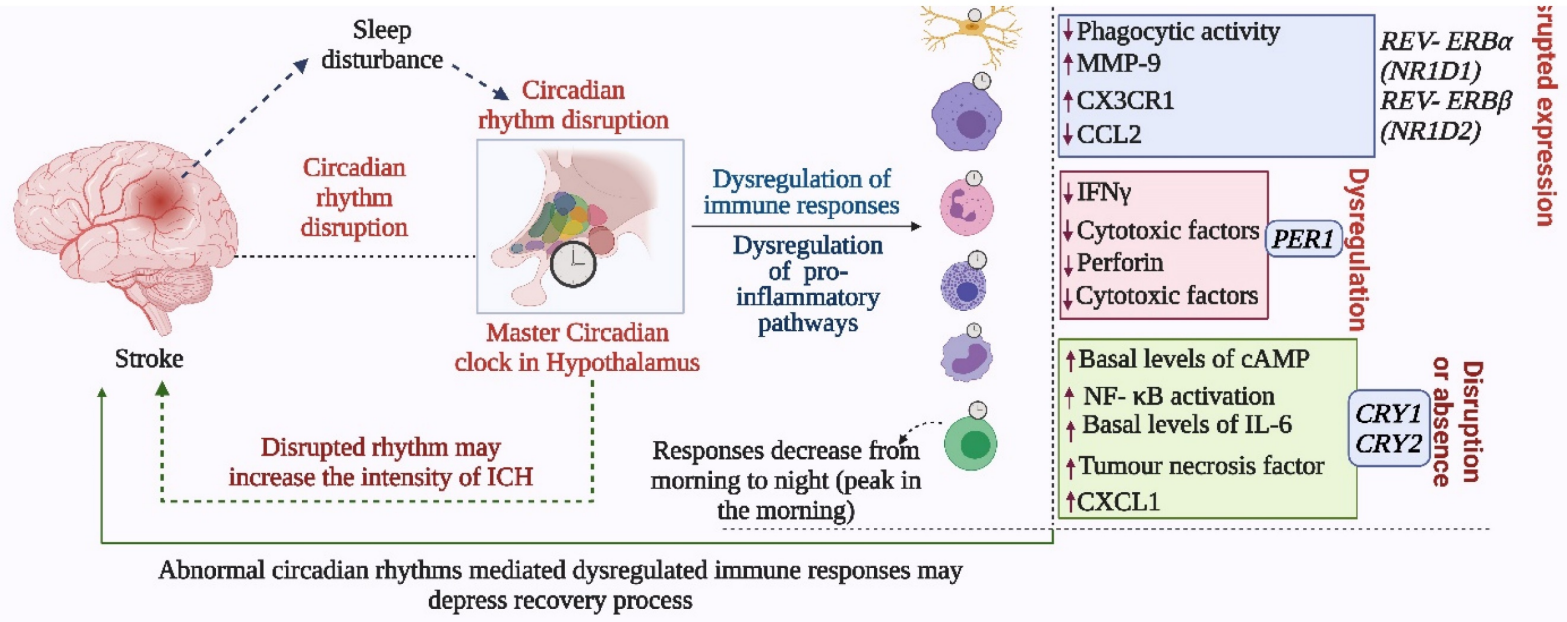
Abnormal circadian rhythm dysregulates immune responses and decreases stroke recovery. The disrupted circadian rhythms in response to environmental cues and stroke impact the normal functions of the immune system, which negatively affects immune responses required for recovery from stroke and prevention of the development of severe symptoms. In addition to the master circadian clock of SCN, the intrinsic cellular clock of immune cells commonly termed intrinsic timers also modulates the function of immune cells. REV- ERBα (NR1D1) and REV- ERBβ (NR1D2) suppress the expression of CX3C- chemokine receptor 1 (CX3CR1) and MMP-9, while REV- ERBα plays a role in the regulation of inflammatory responses and CC- chemokine ligand 2 (CCL2). The absence of CRY1 induces the activation of protein kinase A (PKA) to regulate NF- κB activation, while CRY1/2 is linked with the regulation of IL-6, tumor necrosis factor, and CXCL1 in fibroblasts and macrophages. Moreover, the 24-hour rhythms generated by the master circadian clock in addition to intrinsic clocks modulate T cell mediated immune responses, which peak early in the morning and decrease as the day proceeds toward night. Such regulation of immune responses by the circadian clock defines the fate of stroke symptoms and recovery.

Innate lymphoid cells (ILCs), which produce cytokines such as interleukin (IL)-5 and IL-13, are enriched in the expression of circadian clock-related genes and exhibit diurnal oscillations in response to light cues. BMAL1 deficient ILCs have impaired expression of PER3 and NR1D1, reduction in their selectivity, upregulation of RORγt-dependent target genes, and elevated proapoptotic pathways 39. Wang et al. found circadian oscillations in the expression of IL-22 and IL-17 by ILC3s, which were affected by disruption in the normal clock. REV-ERBα-deficiency reduced ILC3 cell numbers and IL-22 production; however, IL-17 secretion was paradoxically increased. They concluded that ILC3s are regulated by circadian rhythm, whereby the clock regulator REV-ERBα affects ILC3 development and functions because of its association with RORγt 40. These results indicate that circadian regulation is required for the homeostasis of ILCs (Figure 4).

**Figure 5:**
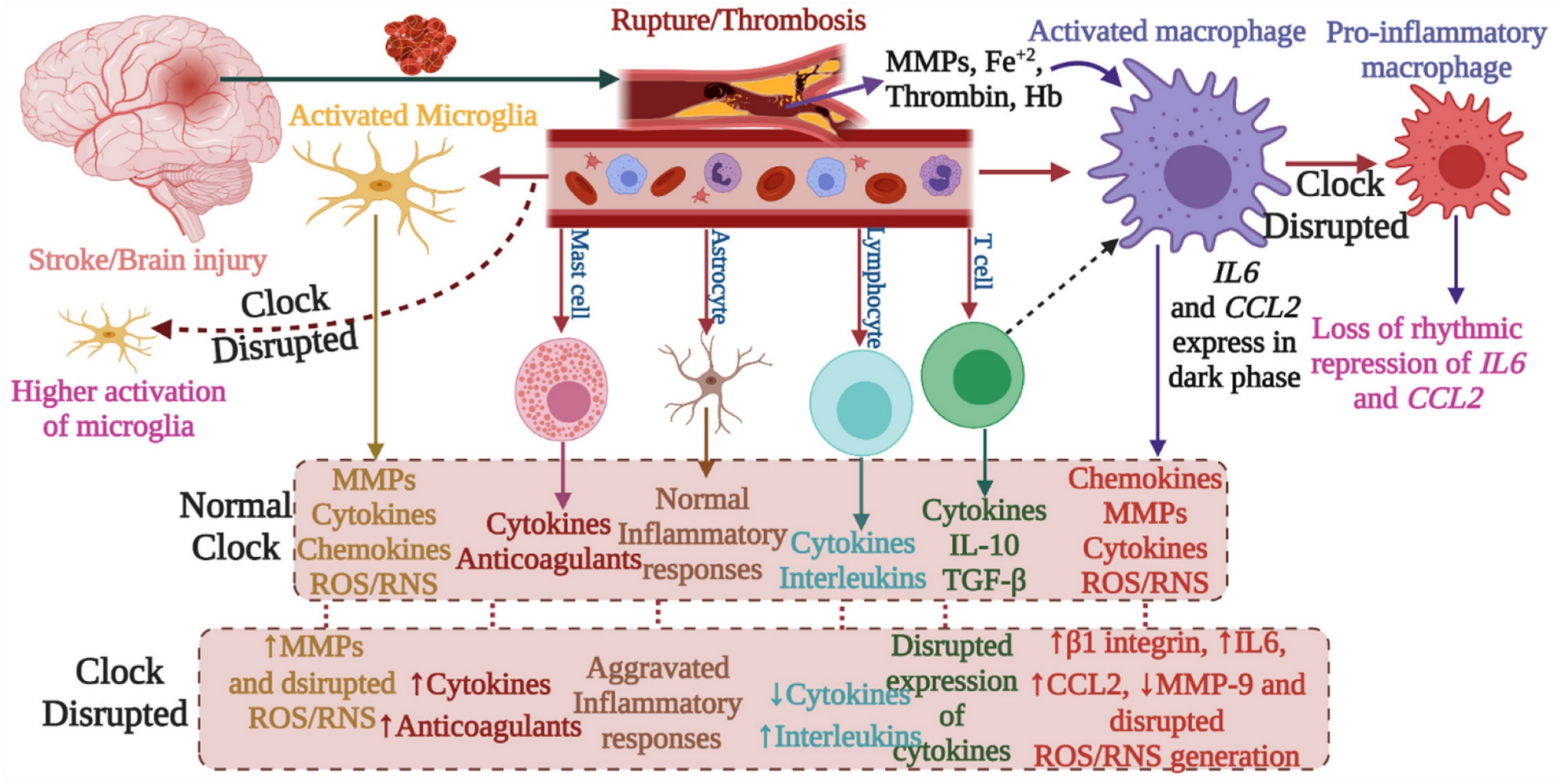
This figure depicts the involvement of circadian rhythm in stroke mediated immune responses. Stroke brain injury results in the activation of macrophages and microglia. These cells regulate immune processes by producing MMPs, cytokines, chemokines, REACTIVE OXYGEN SPECIES, and RNS species. Other immune cells including mast cells, astrocytes, lymphocytes, and T cells are also engaged. These immune responses are regulated by the circadian clock. Clock deficiency or dysregulation leads to altered immune activity; for example, the expression of IL6 and CCL2 loses rhythms, and this results in pro-inflammatory macrophages. Similarly, the disrupted clock causes higher activation of microglia and dysregulates the activities of other immune cells. Such alterations affect the outcome of stroke including its recovery.

T cells produce particular cytokines to activate macrophages in cell-mediated immunity and to promote wound healing. Autoreactive T cells in the CNS are considered to be important in the pathogenesis of stroke 4, while regulatory T cells, producing IL-10 and TGF-β, are crucial for the maintenance of homeostasis of the immune system. T cells are highly dependent on circadian regulation; Nobis et al. assessed the circadian transcriptome of CD8 T cells and found that genes and pathways related to T cell activation are enriched in the daytime, and would facilitate the response to vaccination during that period 41. Oligodendrocytes that are responsible for myelin production possess intrinsic clocks (cellular clock that regulates the physiological activities of cells as well as the release and expression of different molecules) that regulate their activities. Thus, circadian clock-mediated regulation of oligodendrocytes may regulate myelinogenesis during stroke recovery, but this has not been assessed. Mast cells modulating the adaptive immune response (Figure 4-5), can store vasoactive substances such as histamine, cytokines, proteases and anticoagulants 42, which could be regulated by intrinsic cellular clock for better immune reactivity.

Neutrophils store proinflammatory molecules including NADPH oxidase, MMP-8, and MMP-9. They transmigrate into damaged tissue after cerebral ischemia 42 and contribute to brain injury 27. Their functions and concentration in the blood are under circadian control. Circadian clock genes *PER2* and *BMAL1* can systemically regulate the diurnal changes in neutrophil activity, indicating that both the intrinsic cellular clock and master circadian clock (regulates physiological and behavioral activities, and coordinates other biological clocks for synchronization) of SCN can impact the functions of neutrophils. The migration of neutrophils into tissues is influenced by endogenous rhythms due to the circadian expression of adhesion proteins 36. Moreover, stimulation with the toll-like receptor 4 (TLR4) ligand, lipopolysaccharide (LPS), changes serum levels of CCL2, CCL5, CXCL1, IL‑6, and IL‑12, regulated by the expression of *Bmal1* and *Nr1d1* 43.

## Clock genes modulate stroke-mediated immunological responses

Circadian rhythm sleep disorders promote neurodegeneration by inducing microglia-mediated neuroinflammation and protein aggregation, thereby increasing the risk of stroke 38. The intrinsic timers of the circadian clock affect the function of immune cells such as phagocytic activity of macrophages, cytokine release, histamine release, and allergic reactions 44. In the absence of *PER1*, the rhythmic expression of stroke-regulating molecules such as IFNγ, cytotoxic factors, perforin, and granzyme B is disrupted in NK cells 45. While, in macrophages, REV- ERBα (*NR1D1*) and REV-ERBβ (*NR1D2*) suppress the expression of stroke-linked molecules such as MMP-9 and disrupt the regulation of chemokine receptor 1 (Cx3cr1) 44. We know that upregulation of IL-6 on glial cells or neurons in stroke can reflect passive passage and systemic release due to disruption of the blood-brain barrier. It is thought that IL-6 contributes to the regulation/control of oxidative stress and angiogenesis 23,46. CCL2 contributes to post-stroke neurological repair and delivery of various cells into the brain 46. Both IL6 and CCL2 lose rhythmicity in response to clock disruption resulting in the generation of pro-inflammatory macrophages, which increase the severity of stroke 45,47,48. These observations indicate that REV-ERBα modulates the inflammatory functions of macrophages via direct regulation of MMP-9, IL6, and CCL2 49,50. In the absence of *Cry1,* basal levels of cAMP increase to stimulate protein kinase A (PKA) which in turn activates pro-inflammatory factor “NF-κB” 44,51. Moreover, the absence of *CRY1/2* increases the basal levels of IL-6, tumor necrosis factor, and CXCL1 in macrophages 44, indicating that clock genes are involved in the regulation of the molecular effectors of pro-inflammatory pathways, which are largely associated with the development and severity of stroke 51,52. Overall, the functional clock can modulate the development and recovery processes of stroke by regulating the daily flux of inflammatory cells between bone marrow, blood, and immune organs; leukocyte numbers in blood show time-of-day-dependent variation which can be abolished by deletion of the core clock gene *BMAL1*/*ARNTL* or chronic SCN arrhythmia (Figures 4-5) 45,52.

Like the innate immune system, the adaptive immune system is also associated with the development and severity of stroke and is under circadian control 24,35; T and B cells contain circadian oscillations 44. For instance, T cell-mediated immune responses peak early in the morning and decrease as the day proceeds toward night, while the rhythm of T cell counts depends on glucocorticoid, chemokine receptor rhythms, and adrenergic regulation of intrinsic clock 41,44. However, *BMAL1* deficiency negatively affects the development of B cells, which contributes to the adaptive immune response to stroke, the pathogenesis of stroke, and endogenous protection from stroke injury 44,53. Overall, T and B cells exhibit strong circadian oscillations in the blood which are linked with oscillations in CXCR4 and CX3CR1 expression, regulated by glucocorticoids and catecholamines as well as hypoxia-inducible factor 1α signaling 44, which in turn contribute to development and severity of stroke, recovery from stroke, and neuronal protection after brain injury 44,53.

### Treating or correcting dysregulated circadian rhythms in stroke

The understanding of the interaction of molecular components of the clock and critical elements of inflammatory pathways offers the potential for the use of pharmacological agents that target the clock proteins as anti-inflammatory agents. Although the production of high-efficiency molecules that enhance the activity of clock proteins is a challenge, several compounds have been developed that show the benefits of pharmacological modulation of clock proteins. SR9009 is a synthetic REV-ERB agonist that decreases atherosclerotic plaque burden while a synthetic REV-ERB antagonist (SR8278) has detrimental effects on encephalitis. KL001 (stabilizes CRY proteins) has anti-inflammatory effects on fibroblast-like synoviocytes 44.

**Figure 6:**
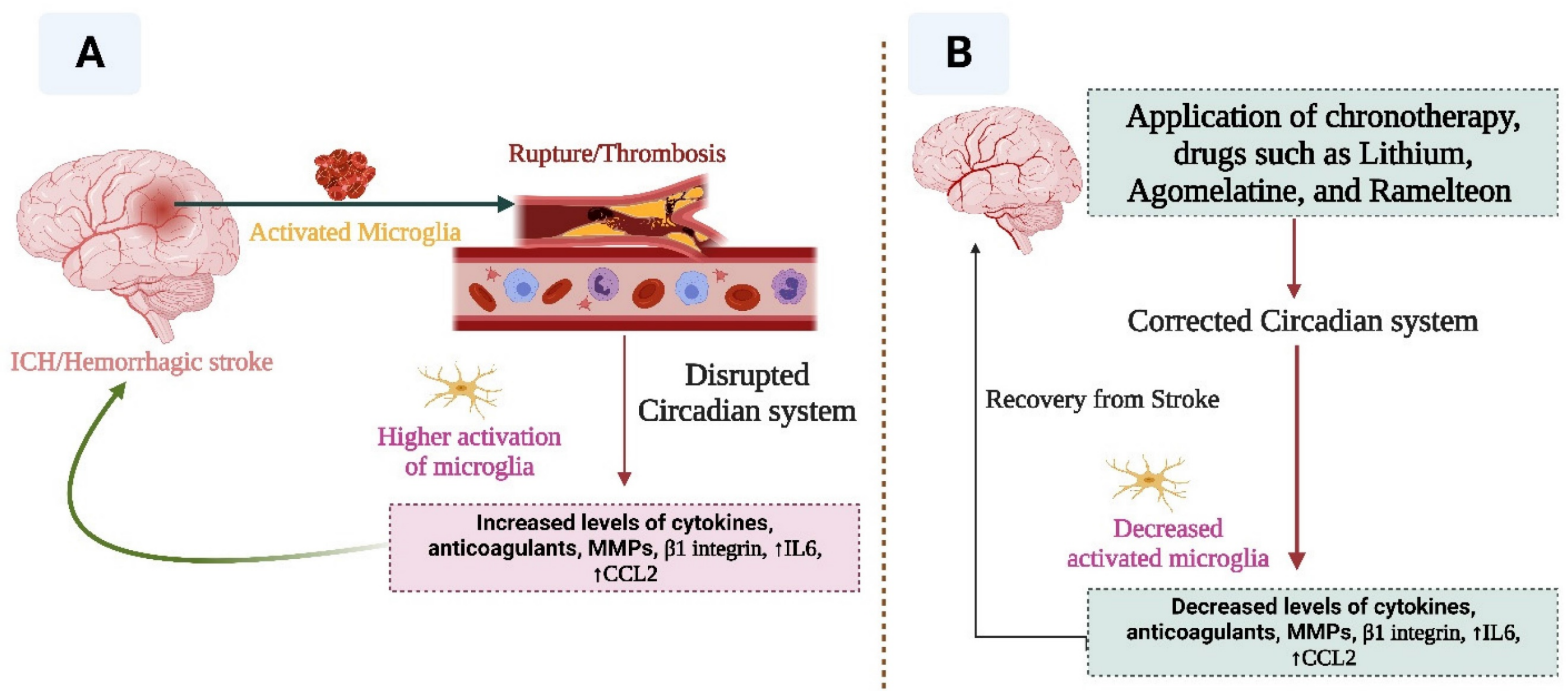
This figure indicates the relationship between stroke and circadian rhythm. Disruption of circadian rhythm facilitates the progression of stroke (A), while correction of the altered circadian system (B) may help in the recovery from stroke.

Recent reports suggest that lithium may contribute to correcting dysregulated circadian rhythm as it can delay sleep-wake phase rhythms and increase social rhythm stabilization. Lithium affects the expression of circadian genes such as *NR1D1*, *PER2*,* and ARNTL*, and promotes melatonin release, thereby enhancing sleep efficiency and increasing sleep duration. One of the most important pharmacological interventions against dysregulated circadian rhythm is melatonin agonists, which can stabilize and regulate normal rhythms (Figure 6). Melatonin administration induces sleep and phase shifts in the circadian clock, and inhibits the drive for wakefulness emanating from the circadian pacemaker; therefore, exogenous melatonin can act as a chronobiotic agent 54. For example, Agomelatine (a melatonin receptor agonist) contributes to the stabilization and regulation of circadian rhythm. Moreover, a melatonin agonist “ramelteon” enhances sleep by affecting melatonin receptors in the SCN. Norris et al. 55 reported that patients who received ramelteon experienced improved sleep. Therefore, ramelteon or analogs should be developed in order to provide better options for correcting dysregulated rhythms in diseases.

As circadian rhythms are directly impacted by light-dark cycles, the most suitable option for correcting dysregulated circadian rhythms can be the pharmacological interventions mentioned above, plus light or dark phase adjustment. Virtual darkness or lightness may be used to treat circadian rhythm disruption. Intrinsically photosensitive retinal ganglion cells (ipRGCs) suppress the action of melatonin, therefore, intervening in the light intake of ipRGCs may stabilize circadian systems in patients with dysregulated circadian rhythms. As ipRGCs are specifically responsive to blue light 56, the treatment that blocks blue light (e.g. virtual darkness therapy) may improve sleep and related conditions in stroke patients.

## Conclusion and perspective

The precise role of circadian clocks in stroke and related cerebrovascular diseases needs to be addressed in future studies. Conditional knockout of specific circadian clock genes, such as within immune cells, would provide an opportunity to test the link between the circadian clock and cerebrovascular diseases/stroke. Also, investigating the molecular mechanisms that underlie the association between circadian rhythms and stroke would be an important step toward developing specific therapeutic strategies. Overall, investigations related to stroke and circadian rhythms as well as their common features (immunity and diseases) may help in developing therapeutic options, alleviate the severity and intensity of stroke symptoms, and prevent post-stroke psychiatric and related disorders.

Disruption in circadian rhythm is linked with various neurological conditions, and cerebrovascular disorders such as stroke are among the major neurological problems in society. Key regulatory elements and immune cells involved in the development of stroke and recovery from stroke are largely controlled by the circadian system. In addition, both circadian rhythms and cerebrovascular disorders (stroke) are linked with depressive disorders as well as sleep-wake cycles/ durations. Overall, these cross-connections suggest that cerebrovascular diseases (stroke) and circadian clocks may be interconnected. It has been implicated as an important lifestyle factor that increases the risk of developing cardiovascular disease, cancer, diabetes, obesity, and cancer. Indeed, circadian clock-mediated chronic sleep and neurological complications increase the risks of stroke. Although stroke has been found to destabilize the circadian rhythms, the relationship between circadian disruption and stroke requires a wide range of investigations. Since the disruption of circadian clocks affects the regulation of many diseases, pharmacological modulation of the circadian clock machinery might provide effective treatment options against cerebrovascular disorders (stroke) and contribute to recovery from stroke.

## Abbreviations

ICH: Ischemic Stroke
CNS: Central nervous system
SCN: Suprachiasmatic nuclei
NK: Natural killer
ipRGCs: intrinsically photoreceptive retinal ganglion cells
MMPs: matrix metalloproteinases
